# Supplementary figures and images for: Diagnosis of soil-transmitted helminth infections with digital mobile microscopy and artificial intelligence in a resource-limited setting
Source: PLoS Negl Trop Dis. 2024 Apr 11;18(4):e0012041. doi: 10.1371/journal.pntd.0012041 (PMC11008773; doi:10.1371/journal.pntd.0012041)

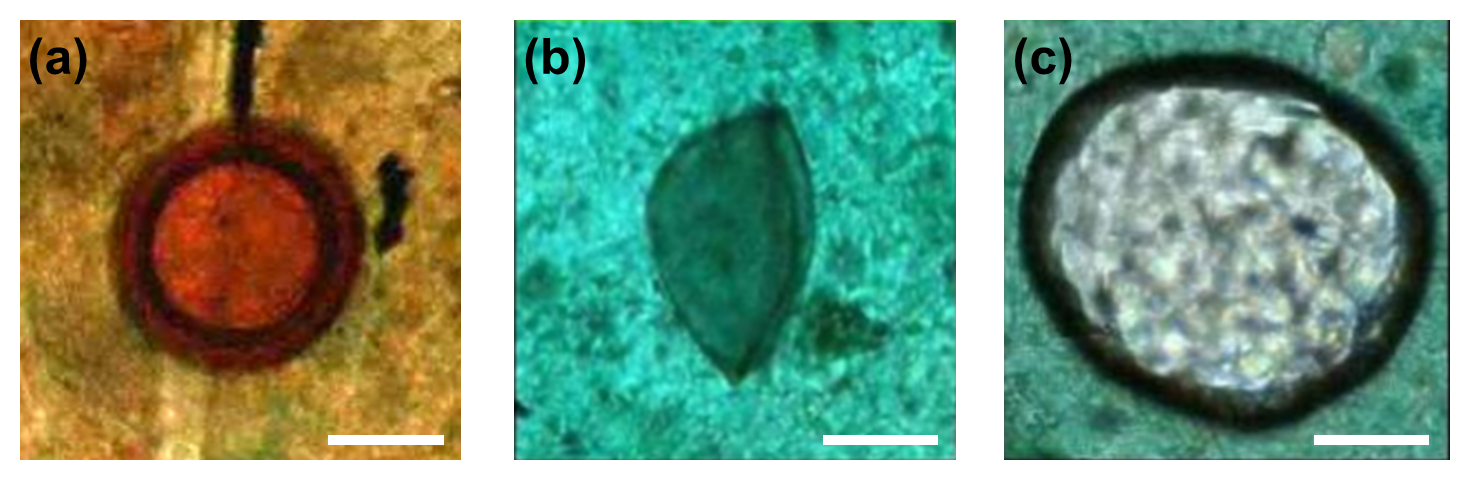

Supplement: S1 Fig — Examples of artefacts classified as parasite eggs by the deep learning system causing a false positive result: (a) A. lumbricoides, (b) T. trichiura, and (c) hookworm (c) findings. (TIF) [file pntd.0012041.s001.tif]

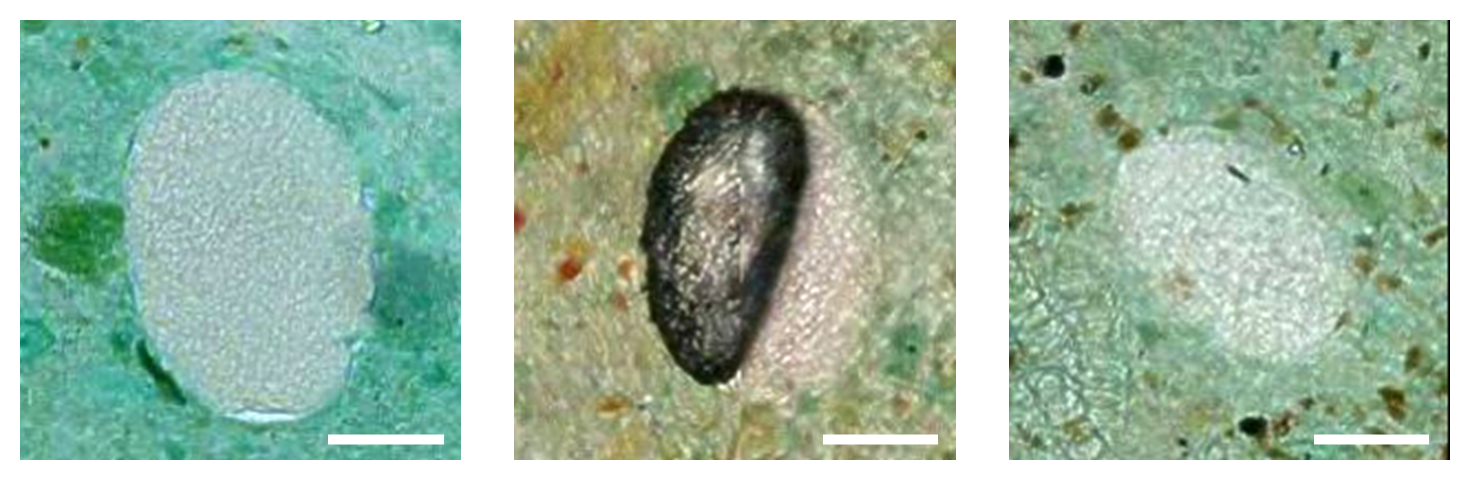

Supplement: S2 Fig — Examples of desiccated hookworm eggs found in the digital images causing false negative results in the deep learning system (DLS) analysis. (TIF) [file pntd.0012041.s002.tif]

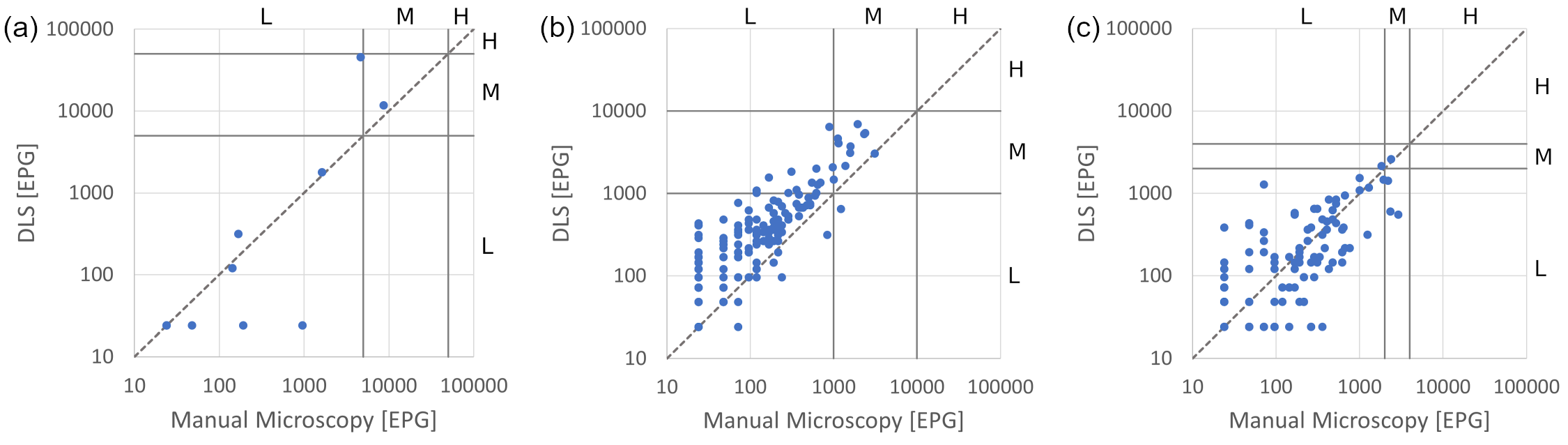

Supplement: S3 Fig — The x-axis represents manual microscopy results, while the y-axis depicts the DLS results, separately for a) A. lumbricoides, b) T. trichiura, and c) hookworm. Infection intensity levels for each species are denoted by letters on the plots (L for light, M for moderate, and H for heavy infection). (TIF) [file pntd.0012041.s003.tif]
