# Supplementary material for: Diagnosis of soil-transmitted helminth infections with digital mobile microscopy and artificial intelligence in a resource-limited setting
Source: PLoS Negl Trop Dis. 2024 Apr 11;18(4):e0012041. doi: 10.1371/journal.pntd.0012041 (PMC11008773; doi:10.1371/journal.pntd.0012041)
Supplement: S1 Protocol — (DOCX) [file pntd.0012041.s004.docx]

**S1 Protocol**

Standard operation procedure for preparation of Kato-Katz samples

For the study “Diagnosis of soil-transmitted helminth infections with digital mobile microscopy and artificial intelligence in a resource-limited setting”

**Preliminaries**

1 Samples should be collected in dry clean containers preferably polypots. They should be fresh or stored at temperatures 23-28 degrees Celsius. The plates, slides, sieves, and soaked cellophane papers should be ready on the bench. The plate should be placed on the slide ready for sample handling.

**Method**

2

Preparation

2.1 Using a spatula, mix the stool sample so that the eggs are evenly distributed in the sample (if present).

2.2 Take approximate 1g of stool and smear on the inside of the wire sieve and continue to smear in a grinding manner till some of the sample pass through the mesh.

2.3 Using the spatula, take a small sample from the bottom surface of the sieve and fill the plate hole by smearing.

2.4 Pass the side of the spatula over the template to remove excess stool material from the edge.

2.5 Remove the template carefully so that the cylinder of stool is left on the slide.

2.6 Gently uplift the plate and discard into the disinfectant solution. Do the same to the sieve.

2.7 Place a piece of cellophane paper soaked in Kato's solution on the specimen portion on the slide. The paper serves as s the cover slip.

2.8 Place the slide upside down on a filter paper on the bench and press gently but firmly to spread the specimen between the slide and the paper. Turn the slide to the upside position. Allow the slide to stand for 30-60 min.

2.9 Place the slide on the microscope and count all the ova within 30-60 minutes.

Counting

3 Counting

3.1 Using x10 objective focus on the specimen and count all hookworm within the first hour.

3.2 In the second round, count all ova and calculate the number of eggs per gram.

3.3 Using the 41.7 mg plate multiply by 24 to get EPG eggs per gram of stool and report.
