# Supplementary material for: Diagnosis of soil-transmitted helminth infections with digital mobile microscopy and artificial intelligence in a resource-limited setting
Source: PLoS Negl Trop Dis. 2024 Apr 11;18(4):e0012041. doi: 10.1371/journal.pntd.0012041 (PMC11008773; doi:10.1371/journal.pntd.0012041)
Supplement: S1 Table — CI = confidence interval. (DOCX) [file pntd.0012041.s005.docx]

**S1 Table**

|  | Sensitivity, % (CI95%) | Specificity, % (CI95%) | Positive predictive value, % (CI95%) | Negative predictive value, % (CI95%) |
| --- | --- | --- | --- | --- |
| Deep learning system |  |  |  |  |
| *A. lumbricoides* | 78.9  (54.4 - 93.9) | 98.6  (97.5 - 99.3) | 57.7  (36.9 - 76.6) | 99.5  (98.7 - 99.9) |
| *T. trichiura* | 92.7  (88.6 - 95.7) | 99.1  (97.9 - 99.7) | 97.7  (94.8 - 99.3) | 97.0  (95.3 - 98.3) |
| Hookworm | 78.4  (71.1 - 84.7) | 97.3  (95.8 - 98.4) | 87.6  (80.9 -92.6) | 95.0  (93.0 - 96.5) |
