# Supplementary material for: Diagnosis of soil-transmitted helminth infections with digital mobile microscopy and artificial intelligence in a resource-limited setting
Source: PLoS Negl Trop Dis. 2024 Apr 11;18(4):e0012041. doi: 10.1371/journal.pntd.0012041 (PMC11008773; doi:10.1371/journal.pntd.0012041)
Supplement: Attachment — Submitted filename: Review_TEA.pdf [file pntd.0012041.s008.pdf]

Diagnostics of soil-transmitted helminths with digital mobile microscopy and artificial intelligence in a resource-limited setting

Lundin et-al, reports a digital mobile microscopy integrated with Artificial Intelligence for the quick detection of stool parasites. Soil Transmitted Helminths is indeed a big challenge in Low Medium Income Countries. Affordable, easy-to-use diagnostic tools would significantly contribute to the control and elimination programs. The paper proposes a digital imaging platform that is superior in performance to the reference manual microscopy commonly used. The reported accuracy for detection of STHs with the Deep Learning System is promising.
The submitted manuscript is well written and quite readable as well. However, a few remark that could potentially improve the quality of the paper is stated below:

If these issues are addressed, I recommend the paper for acceptance.

This seems to me like a piece of engineering work. I struggle a bit to find the scientific contribution of this work. Several research groups are working on developing AI-based digital pathology for Neglected Tropical Diseases. While the introduction highlights the application of image-analytical methods to microscopy diagnostics, the scientific challenge/gap is still unclear. Previous work by Ward et-al is highlighted below. I will expect a clear contribution of the authors work to existing similar approach.

Ward P, Dahlberg P, Lagatie O, Larsson J, Tynong A, Vlaminck J, et al. (2022) Affordable artificial intelligence-based digital pathology for neglected tropical diseases: A proof-of-concept for the detection of soil-transmitted helminths and *Schistosoma mansoni* eggs in Kato-Katz stool thick smears. PLoS Negl Trop Dis 16(6): e0010500. https://doi.org/10.1371/journal.pntd.0010500

The equipment and setup used for the digitization of microscopy slides in a primary health care setting listed a laptop computer used to operate the whole slide scanner and manage digital slides. I wonder why an online computational procedure is necessary. The laptop should have sufficient computational capacity to implement the developed AI models offline. This will deal with the challenge of the availability of mobile data networks in remote areas where such devices are needed the most. Authors motivate and justify why the DLS is not implementable on the laptop which is already part of the equipment set-up. A deepLabV3-MobileNetV3 deep learning model has been implemented on a Raspberry Pi 4B with Coral USB accelerator as reported by Oyibo et-al. This further re-emphasize the endless possibilities of implementing DLS on general computers. An online DLS systems does not seem to add value except the authors could justify this in their work.

Prosper Oyibo, Brice Meulah, Michel Bengtson, Lisette van Lieshout, Wellington Oyibo, Jan-Carel Diehl, Gleb Vdovine, Temitope E. Agbana, "Two-stage automated diagnosis framework for urogenital schistosomiasis in microscopy images from low-resource settings," J. Med. Imag. 10(4) 044005 (7 August 2023)https://doi.org/10.1117/1.JMI.10.4.044005

Digitization of Slides
How many fields of view are required to cover the entire sample. I can imagine that with a 20x objectives, there is gain in resolution but decrease of the field of view. This would translate to the need for more images and increased processing time as well. Author should comment on why a low magnification is not used?

The end-to-end sample scanning, upload and processing time is still unclear. Author should clearly state the total time from sample insertion to result output. Is there a gain in time as compared to reported existing

techniques. There are existing offline methods that proposes a turnaround time of 15 minutes. What is the value addition of this work in that regard?

Data annotations requires some expert opinion, are the researchers experts in this field? Could this be well stated?

An analysis speed of approximately 14 digital samples per hour seems to be quite slow. What is the plan to increase the processing speed of the samples?

The parameters of the equations provided in line 346 and 347 are not clearly explained. Authors should explain these parameters accordingly.

What is the limitation of the study?
