# Supplementary material for: Diagnosis of soil-transmitted helminth infections with digital mobile microscopy and artificial intelligence in a resource-limited setting
Source: PLoS Negl Trop Dis. 2024 Apr 11;18(4):e0012041. doi: 10.1371/journal.pntd.0012041 (PMC11008773; doi:10.1371/journal.pntd.0012041)
Supplement: Attachment — Submitted filename: Review of the manuscript.docx [file pntd.0012041.s006.docx]

**Review of the manuscript: Diagnostics of soil-transmitted helminths with digital mobile microscopy and artificial intelligence in a resource-limited setting**

Summary: Lundin et-al proposes an interesting automation of manual microscopy by the integration of digital scanner and an online image analysis AI pipeline that identifies the target parasites in the registered images. The method, if successful could provide quick diagnosis of soil transmitted helminths in resource limited settings. This will bridge the diagnostic gap of lack of expertise, high cost of training lab technicians, affordability of equipment and scarcity of diagnostic tools where they are mostly needed. The system was validated on the field and the performance metrics measured with respect to sensitivity and specificity include - Sensitivity and Specificity. The authors claim that the proposed system performed relatively better than the reference standard (microscopy) in terms of sensitivity and specificity.

Introduction: The introduction is well written in nice English and well referenced. Some challenges observed are listed below. I believe if the manuscript is reviewed with the challenges sorted out, the manuscript should be accepted as a contribution to the community.

**Observed challenges with the introduction section of the manuscript.**

- A quick summary of the portable digital devices developed for the detection of STH in recent past, however, the limitations of this techniques and how the newly proposed method mitigate against the identified gaps is not clearly stated. I therefore miss the scientific contribution of this work. I do not see the gap the proposed solution is trying to bridge. It is more of an engineering solution report than a scientific paper as it is. Authors should clearly state what the limitations of the already developed portable devices digital optical platforms for diagnosis and explain how their system mitigate these limitations. State the novelty of your work.
- Offline data processing technique has its good advantages. You could leverage on sufficient computational power etc. However, wide availability of internet data to connect the isolated system to a remote server is still a challenge in remote areas where such devices would be deployed. With the availability of miniaturized general computer processors such as Raspberry pi, Jetson Nano, and other computers that could be integrated into the device, one may argue that the need for an online transfer of data is unnecessary and as such add no significant value. Could the authors justify why a remote processing of acquired digital data is superior and better than including a local low-cost hardware (<€100) in an integrated automated system that could be used offline. Data driven algorithms with minimal computational complexity implementable on this low-profile general computer can be developed.
- How is the data uploaded online? File transfer server? Customized proprietary software?

Questions about the Ethics Statement:

- Who offered the treatment? Any follow up on treated infected patient? Was the local/National NTD Program management included in the treatment for effective follow up?

Questions about the Data acquisition and preparation

- Since samples were collected between (March - October) approximately 8 months period, it is unclear how the samples were stored. Were the samples collected processed and examined in batches or were they all stored and processed at once? Authors should please clarify.
- It was stated that slides were reviewed by a laboratory technologist, but to compare the result of the manual microscopy with the performance of the AI, Standard protocol require the independent evaluation of the samples by two microscopist and a 3^rd^ independent microscopist in case of discrepancy in the results of the first and second microscopist? It is not clear to me if the authors have followed this protocol. Authors should justify the use of one lab technologist and state the discrepancy threshold selected to motivate the use of a 3^rd^ microscopist if two microscopists were used and how the protocol was executed.

**Digitization of Slides**

- The specification of the scanner is not clear: Why is coarse focusing necessary? Why cannot full time autofocusing routine be realized? How much time is required to achieve the average coarse focusing? How is this different from manual microscopy.
- How many digital images are registered per sample? What is the size of each of image registered by the optical system? The author is not clear or specific about turn around upload time. How long is required to upload images registered from an image. I can imagine that if a 10x objective is used, then a full scan of a Kato Katz sample would result in the registration of over 350 digital images? Therefore a scan of over a 1,000 samples should result in over 350k digital images to be uploaded. Author should state this clearly so that reader may gain sufficient insight into the operations of the system.
- How is the upload done practically? Dedicated File transfer protocol?

**Training of the deep learning system**

- How were the annotations made? Any validation and confirmation by an expert?
- Author reports 14 sample analysis in 1 hour. This implied that one sample is analyzed on the AI platform in about 4.3 minutes. Is this the time indeed required for analysis on the online platform for each sample? What is ended to end time required for sample scanning, autofocusing and data processing to spit out the results? Any plan to speed up this processing time?

**Statistical Analysis**

- Explanation of the sample size calculation formular should be included in the Study design

**Results**

- Author should please describe the representations of TT, AL etc in Tab. 3. Table not as clear without the description of those abbreviations.
- How were the digitized samples independently reviewed? By the same lab technician? Will this not introduce a bias to the result if this is the case? This is not clear stated. Author should clarify this accordingly.
- What is responsible for the significant difference in the parasite count of A. lumbricoides and T. Trichiura. It is difficult to accept the manual microscopy manual count if this count has been achieved by one lab scientist. An acceptable scientific protocol is to use two independent lab scientist and take the average of their parasite count estimation. A 3^rd^ lab scientist is often used to check the discrepancy and variation accordingly. Authors should get this sorted accordingly.

**Discussion**

- Can you compare your result to the WHO Target Product Profile and see how your device compares and where it fits? How you optimize your performance so system can be available for use on the field?
- I observe the use of one microscopist is explained and justified in the discussion. I think this should have been done earlier. Somewhere in design and protocol etc so that reader will not jump into conclusion and be biased towards the results obtained in this research work.
- Competing interest statement is not included. Authors should check if this is a requirement and include it accordingly.

Conclusion: I appreciate the work of the authors. I belive this could provide some valid contribution in the NTD space if some of the concerns and challenges stated are being addressed and the manuscript revised.
