# Supplementary material for: Diagnosis of soil-transmitted helminth infections with digital mobile microscopy and artificial intelligence in a resource-limited setting
Source: PLoS Negl Trop Dis. 2024 Apr 11;18(4):e0012041. doi: 10.1371/journal.pntd.0012041 (PMC11008773; doi:10.1371/journal.pntd.0012041)
Supplement: Attachment — Submitted filename: Lundin_et_al_Response_Letter.docx [file pntd.0012041.s007.docx]

**Point-by-point response related to the manuscript “Diagnostics of soil-transmitted helminths with digital mobile microscopy and artificial intelligence in a resource-limited setting” (PNTD-D-23-00949)**

We thank the reviewers for a careful assessment of our manuscript, and we have addressed all issues raised point-by-point below. Line numbers refer to the manuscript version “Lundin_et_al_track_changes_031223”

**Reviewer 1.**

**Reviewer 1.1**: Introduction: The introduction is well written in nice English and well referenced. Some challenges observed are listed below. I believe if the manuscript is reviewed with the challenges sorted out, the manuscript should be accepted as a contribution to the community.

**As a response:** We thank the reviewer for the positive feedback and have addressed the issues raised, point-by point below.

**Reviewer 1.2:** A quick summary of the portable digital devices developed for the detection of STH in recent past, however, the limitations of this techniques and how the newly proposed method mitigate against the identified gaps is not clearly stated. I therefore miss the scientific contribution of this work. I do not see the gap the proposed solution is trying to bridge. It is more of an engineering solution report than a scientific paper as it is. Authors should clearly state what the limitations of the already developed portable devices digital optical platforms for diagnosis and explain how their system mitigate these limitations. State the novelty of your work.

**As a response:** We thank the reviewer for pointing this out and have now clarified how our study differs from previously published studies and methods. We were not able to find any previous studies that would have applied AI-based methods for STHs in a similar basic laboratory in a primary health care setting. Also, the number of slides analysed exceeds previous similar work with a large margin and our study is likely to be the first where the entire Kato-Katz slides were scanned i.e. where true whole-slide imaging was used. We added these statements on lines 23-28 in the Abstract, 115-116 in the Introduction and on lines 520-523 in the Discussion.

**Reviewer 1.3:** Offline data processing technique has its good advantages. You could leverage on sufficient computational power etc. However, wide availability of internet data to connect the isolated system to a remote server is still a challenge in remote areas where such devices would be deployed. With the availability of miniaturized general computer processors such as Raspberry pi, Jetson Nano, and other computers that could be integrated into the device, one may argue that the need for an online transfer of data is unnecessary and as such add no significant value. Could the authors justify why a remote processing of acquired digital data is superior and better than including a local low-cost hardware (<€100) in an integrated automated system that could be used offline. Data driven algorithms with minimal computational complexity implementable on this low-profile general computer can be developed.

**As a response:** We agree with the reviewer that good internet connections might not be available in all rural areas. However, in our studies in rural Kenya and Tanzania, connectivity has been surprisingly good, and upload of data has not been a bottleneck. There are several advantages of data upload to a central repository in a cloud environment, including opportunities to have human experts in the loop for remote verification, to create large-scale databases, continuously monitor the accuracy of the AI and quality of samples, perform surveillance of the target diseases and to collect more training data. Ultimately, as the reviewer points out, the algorithms should also be applicable without internet connectivity and to run on local devices, but also in that case sharing and transferring data when mobile networks are available should be considered an advantage. We added a comment on this in the Discussion on lines 569-579

**Reviewer 1.4:** How is the data uploaded online? File transfer server? Customized proprietary software?

**As a response:** A desktop application (Aiforia Connector, Aiforia Technologies, Helsinki, Finland) was used to convert the files captured by the scanner to a wavelet file format (Enhanced Compressed Wavelet, Hexagon Geospatial, Madison, AL, USA) with a compression ratio (1:16) previously shown to preserve sufficient detail to not alter image-analysis results [32]. The same application automatically compresses the wavelet file into a single zip-file and uploads the file to the image-management and machine-learning platform (Aiforia, Aiforia Technologies, Helsinki, Finland). On the cloud server, the WSIs are stored as JPEG-compressed tiles sized 512x512 pixels with a pyramid-structured image containing all zoom levels. A typical WSI comprises approximately 10,000 tiles. This information was added to the manuscript on lines 235-237, lines 239-240 and lines 249-251.

**Reviewer 1.5:** Questions about the Ethics Statement: Who offered the treatment? Any follow up on treated infected patient? Was the local/National NTD Program management included in the treatment for effective follow up?

**As a response:** The treatment was offered by a designated clinician from the study hospital (Kinondo Kwetu Hospital). There was a follow-up on treated infected patients that was conducted by the Kwale County NTD Program coordinator who also provided the necessary drugs. The National NTD Program management was included in the treatment for effective follow up. This information was added to the manuscript on lines 140-144.

**Reviewer 1.6:** Questions about the Data acquisition and preparation: Since samples were collected between (March - October) approximately 8 months period, it is unclear how the samples were stored. Were the samples collected processed and examined in batches or were they all stored and processed at once? Authors should please clarify.

**As a response:** During the study period, twenty samples were collected per day. They were processed in a batch, examined and results recorded. Remaining stool of each sample was stored in a refrigerator for reference and discarded after a week on a ‘first-in, first-out’ basis. This information was added to the manuscript on lines 180-182 and lines 202-203.

**Reviewer 1.7:** It was stated that slides were reviewed by a laboratory technologist, but to compare the result of the manual microscopy with the performance of the AI, Standard protocol require the independent evaluation of the samples by two microscopist and a 3^rd^ independent microscopist in case of discrepancy in the results of the first and second microscopist? It is not clear to me if the authors have followed this protocol. Authors should justify the use of one lab technologist and state the discrepancy threshold selected to motivate the use of a 3^rd^ microscopist if two microscopists were used and how the protocol was executed.

**As a response:** We agree with the reviewer that ideally the physical samples would have been evaluated by two microscopists and a third in case of discrepancy between the first two. However, since the time between manual microscopy and digital scanning of the sample should be minimized in order to prevent changes in morphology the targets, e.g. hookworm eggs, we decided to engage only a single microscopist. On the other hand, the digital slides were evaluated by four observers as stated on lines 288-289 and discrepancies between manual and digital findings reported in detail on lines 404-406 and lines 410-12. In future studies, multiple manual microscopists could be considered, if the time to digitization can be kept short (less than 30 minutes) or if the slide is first scanned and the subject to manual microscopy. We have commented on this in the Discussion on lines 541-548.

**Reviewer 1.8:** Digitization of Slides

The specification of the scanner is not clear: Why is coarse focusing necessary? Why cannot full time autofocusing routine be realized? How much time is required to achieve the average coarse focusing? How is this different from manual microscopy.

**As a response**: With the instrument used in the current study (Ocus 20, Grundium, Tampere, Finland) course focusing is only needed to be performed on one field-of-view before initiation of the scanning. During scanning the focusing is fully automated. Adjusting the course focus in average takes 5-10 seconds. We have clarified this in the manuscript on lines 228-231.

**Reviewer 1.9:** How many digital images are registered per sample?

**As a response:** The microscope vendor does not disclose exact information on the field of view, scanning parameters, or the overlap of adjacent images during scanning; therefore, it is not clear how many images are recorded. However, digitizing a sample with a size of 25 mm x 25 mm, using a microscope equipped with a 6-megapixel camera and an optical system resulting in a pixel size of 0.48 um/px on the image plane, would require a minimum of approximately 450 images. In practice, the number of images is larger when considering the overlap needed for stitching and the possible cropping of the maximum field of view available to retain better image quality across the field of view. On the cloud server the WSI is stored as smaller (512 x 512 pixels) image tiles and a typical sample consists of approximately 10,000 tiles. Information on this was added to lines 249-251

**Reviewer 1.10:** What is the size of each of image registered by the optical system?

**As a response:** The size of each registered image is not clear. The microscope is equipped with a camera featuring a 6 MP sensor. However, the microscope vendor does not disclose information on how the sub-images of the whole slide image are processed. We also kindly refer to the answer above. Size of the image tiles after upload is 512 x512 pixels. Information on this was added to lines 249-251

**Reviewer 1.11:** The author is not clear or specific about turn around upload time. How long is required to upload images registered from an image.

**As a response:** Turnaround time for uploading of samples was 10–20 min per slide with the mobile network (upload speed 5–8 Mbps) or ADSL connection (upload speed 5–10 Mbps). We have now specified that the time reported on lines 247-249 is per sample.

**Reviewer 1.12:** I can imagine that if a 10x objective is used, then a full scan of a Kato Katz sample would result in the registration of over 350 digital images? Therefore, a scan of over a 1,000 samples should result in over 350k digital images to be uploaded. Author should state this clearly so that reader may gain sufficient insight into the operations of the system.

**As a response:** Here we refer to a previous response above related to slide upload.

**Reviewer 1.13:** How is the upload done practically? Dedicated File transfer protocol?

**As a response:** Please see previous response above and information added on lines 235-237 and lines 238-241.

**Reviewer 1.14:** Training of the deep learning system: How were the annotations made? Any validation and confirmation by an expert?

**As a response:** Initially, a set of 1,339 annotations was generated by manually browsing the whole-slide images and annotating visible findings of *A. lumbricoides* (n=371), *T. trichiura* (n=530), hookworm (n=51), and artefact (n=446). Subsequently, the remaining object-level annotations were carried out using machine learning-assisted annotation. The annotation tool for verifying machine learning-assisted annotations is presented in Figure 4. The initial annotations were collectively performed by researchers (AS, OH, SH, SV). However, due to technical constraints, the remaining annotations, utilizing an AI-assisted method, were completed by researcher AS. In cases of uncertainty, confirmation was sought from other researchers as well. It is acknowledged that this method may not be the most reliable, and this limitation will be addressed in future studies. Clarifications on this has been added on lines 289-290 and lines 294-296

**Reviewer 1.15:** Author reports 14 sample analysis in 1 hour. This implied that one sample is analyzed on the AI platform in about 4.3 minutes. Is this the time indeed required for analysis on the online platform for each sample? What is ended to end time required for sample scanning, autofocusing and data processing to spit out the results? Any plan to speed up this processing time?

**As a response**: The sample scanning process, including autofocusing and image stitching, typically took an average of approximately 5-10 minutes. The compression and upload time for one sample ranged from 10 to 20 minutes, and the analysis took around 5 minutes on average. This resulted in a total end-to-end time of 20 to 30 minutes, on average. We would like to emphasize that the digital diagnostic pipeline in this study is by no means optimized. The most optimal and cost-efficient approach to digital diagnostics needs to be addressed in future studies. We refer to a previous response above related to local versus cloud processing. In this study, we have observed that the cloud solution is technically feasible and realistically deployable. The local deployment of image analysis could further decrease the overall processing time by eliminating image compression and uploading steps. We have added comments on this to the manuscript on lines 231-233, lines 247-249 in the Methods section and on lines 569-579 in the Discussion.

**Reviewer 1.16:** Statistical Analysis: Explanation of the sample size calculation formular should be included in the Study design

**As a response:** The sample size calculation formulas for sensitivity and specificity have been added to the Methods section (Statistical analysis), and the parameters used defined in the text. Briefly, we used the Buderer formula for equivalence of diagnostic tests to calculate the minimum sample size required. The suggested changes have been added on lines 355-360.

**Reviewer 1.17:** Results: Author should please describe the representations of TT, AL etc in Tab. 3. Table not as clear without the description of those abbreviations.

**As a response**: The abbreviations have been replaced by the names of the parasites in Table 3.

**Reviewer 1.18:** How were the digitized samples independently reviewed? By the same lab technician? Will this not introduce a bias to the result if this is the case? This is not clear stated. Author should clarify this accordingly.

**As a response**: Researchers AS, SV, SH and JL reviewed the AI results found in the digital samples in the test set, independently from the lab technologist (FK) who performed the manual microscopy. We have clarified this in the manuscript on lines 404-406

**Reviewer 1.19:** What is responsible for the significant difference in the parasite count of A. lumbricoides and T. Trichiura. It is difficult to accept the manual microscopy manual count if this count has been achieved by one lab scientist. An acceptable scientific protocol is to use two independent lab scientist and take the average of their parasite count estimation. A 3^rd^ lab scientist is often used to check the discrepancy and variation accordingly. Authors should get this sorted accordingly.

**As a response**: Here we refer to our previous response above related to the number of observers. Our assumption regarding the significant difference in parasite counts between manual microscopy and AI is that counting a larger number of parasite eggs in high-intensity infections is not feasible for a human observer, especially with a larger number of slides to be assessed. Our results also suggest that manually microscopy of the physical slide within a typical time window may result in underdiagnosis in some cases. Calculating a large number of findings accurately during the manual process is challenging, and similarly, there is a high risk of missing findings when they are sparse. Comments on this can be found in the Discussion on lines 499-505 and lines 546-548.

**Reviewer 1.20:** Discussion: Can you compare your result to the WHO Target Product Profile and see how your device compares and where it fits? How you optimize your performance so system can be available for use on the field?

**As a response**: We compared our method to the WHO Target Product Profile for STHs and found that it fulfils most of the criteria for a laboratory-based test: Our method can be used in regional or national diagnostic testing laboratories by trained laboratory technicians (< 1-week training); is portable (weighs less than 5 kg including a laptop computer and router), is specific (≥ 94%) to each Ascaris, Trichuris and hookworm and has a sensitivity of at least 60% for each of the three species, if the combined manual microscopy and visual analysis of digital slides is used as the reference standard. If run on battery, the method also has the potential to be used as a point-of-sampling test, where health personnel and community health workers should be able to perform and interpret the test with only a single day of training. Further, the TPP test for point-of-sampling should allow for a throughput of at least seven samples per hour and its cost should not exceed 3 USD per test. Further studies are needed to assess if our method could meet these requirements. We added this text to the Discussion on lines 525-539

**Reviewer 1.21:** I observe the use of one microscopist is explained and justified in the discussion. I think this should have been done earlier. Somewhere in design and protocol etc so that reader will not jump into conclusion and be biased towards the results obtained in this research work.

**As a response**: We refer to previous comments related to this and have added a comment to the Methods section on lines 194-196, in the Supporting Protocol and Discussion on lines 551-552.

**Reviewer 1.22:** Competing interest statement is not included. Authors should check if this is a requirement and include it accordingly.

**As a response:** It seems that the competing interest statement has not be provided to the reviewers. It was added to the system upon submission and reads as follows: Johan Lundin and Mikael Lundin are founders and co-owners of Aiforia technologies Plc. Dr Johan. Lundin and Antti Suutala reported having a patent for Mobile Microscope pending (no.WO2017037334A1; the invention is related to the use of fluorescence imaging filters combined with inexpensive plastic lenses; all rights are with the University of Helsinki) and Johan Lundin having a patent for a slide holder for an optical microscope pending (no.WO2015185805A1; related to motorization of regular microscopes). No other disclosures were reported.

**Reviewer 1.23:** Conclusion: I appreciate the work of the authors. I believe this could provide some valid contribution in the NTD space if some of the concerns and challenges stated are being addressed and the manuscript revised.

**As a response:** We thank the reviewer for a detailed evaluation and hope that the amended manuscript addresses the raised issues.

**Reviewer 2.**

**Reviewer 2.1:** General comments: It is not clear to me where the authors position their invention. Is it to support program-decision making (determine the frequency and intensity of MDA), as highlighted in the introduction, or to support diagnosis in the health center, as concluded in the abstract. Both are not only quite distinct in decision making (population level vs. individual treatment) and field use (point-of-sampling vs. point of care), I also doubt whether Kato-Katz thick smear is the diagnostic standard in a health center. Too often this is just a direct smear, to ensure detection of a wider spectrum of parasites, including protozoa.

**As a response**: The main use of the current method is within settings where Kato-Katz smears are used. These are likely to be within monitoring and evaluation of control programs. We agree with the reviewer that this was not clearly stated and we have now clarified this on lines 51-52 in the Abstract, Author Summary lines 68-70 in the Discussion on lines 593-594

**Reviewer 2.2:** The study design has some an important flaw: the slides were first subjected to manual microscopy, after which they were scanned. Given a time bounded procedure such as Kato-Katz (hookworm eggs disappear), this has some consequences. This might indeed explain why some hookworms were missed by DSL. Yet, it might also explain why some Ascaris and Trichuris eggs were detected by DSL but not by manual microscopy. While hookworm eggs disappear over time due to the stool clearing process, Ascaris and Trichuris eggs might become more visible over time. In other words, the improved accuracy might be solely due to a difference in clearing time. This is in particular when slides were immediately examined (line 165), and hence there is slides were not cleared at all. Study cannot be redone, but this should be explicitly mentioned in the discussion.

**As a response:** We thank the reviewer for this important observation and have added comments on this to the Discussion on lines 499-505 and 551-552.

**Reviewer 2.3.1:** I feel that result section misses some clarity/info. First, the word ground truth in an AI setting for manual reading is confusing. My interpretation of ground truth is that whole digitized slides are manually and independently annotated by preferable multiple independent experts. Ground truth is now used for manual microscopy (Table 3).

**As a response**: We agree with the reviewer that the term ground truth is not optimal, given that manual microscopy was performed by a single observer. We have therefore replaced the term “ground truth” with “reference standard” throughout the manuscript. Related to the use of a single observer for manual microscopy we also kindly refer to comments to questions raised by Reviewer 1 above.

**Reviewer 2.3.2:** Second, I would also propose to add a Table that represents that results after visual review of the digitized samples. This will help the reader to reasoning of the conclusions about the sensitivity/specificity. A sensitivity of 70 – 92% compared to manual microscopy is not really an improvement, whereas the visual review indicates that manual microscopy missed most of the positive samples detected by DLS.

**As a reponse**: We have added a Supporting Table 1 to the Results, presenting the sensitivity and specificity following the visual review of the digitized samples and text in the Results on lines 410-412, lines 415-417 and referring to the new table S1Table.

**Reviewer 2.3.3:** Third, I would welcome a scatter plot plotting the EPG for manual microscopy as a function of those obtained by DLS that could replace Table 4. Such a graph would give more insights in the data: (i) correlation in EPG between both

diagnostics; (ii) where they fail compared to the other one (dots on the y-/x- axis), (iii) agreement in classification of infection intensity, and (iv) highlight the visual review results (this might require some thought).

**As a response**: A scatter plot (S3 Fig) presenting the requested EPG results have been added to the Supporting Material and a comment in Results on lines 450-452.

**Reviewer 2.4**: Appreciate the attention the authors give to report according to the STARD-guidelines. Given the involvement of AI, I would encourage them to also consult the STARD-AI guidelines (Sounderajah et al., 2021; BMC OPEN Protocol), and to adapt the manuscript accordingly.

**As a response**: We consulted the mentioned article by Sounderajah, and doublechecked our manuscript for compliance. According to the article the official STARD-AI checklist is yet to be published, but our article should be compliant with all items mentioned in the protocol by Sounderajah et al (i.e. computational environment, description of software, version of deep learning algorithm, hardware used for training etc). We clarified some of the terminology to match the terms used in the original STARD checklist i.e. “index test” and “reference standard”.

**Reviewer 2.5:** Author summary: propose to use more lay language. For example, intestinal worms instead of STHs.

**As a response**: We added the term intestinal worms to the Author summary on line 55 for a more lay language, but would prefer to also keep the term soil-transmitted helminths in order to be more specific.

**Reviewer 2.6**: Methods: Line 121 and 122; do we need the abbreviations TUM and NACOSTI – they are only used once.

**As a response**: We removed the abbreviations accordingly.

**Reviewer 2.7:** Line 128: no need to capitalize albendazole

**As a response**: We changed this accordingly.

**Reviewer 2.8:** Line 134: please refer to STARD-AI (see general comment) and adapt the manuscript were required.

**As a response**: Please see previous response above, related to STARD-AI.

**Reviewer 2.9:** Line 139: there were any inc/exclusion criteria

**As a response**: We thank the reviewer for this important observation and have added inclusion and exclusion criteria to the Study design on lines 161-164

**Reviewer 2:9:** Fig 1: see STANDARD AI

**As a response**: Please see previous response above related to STARD-AI, terminology updated in Fig 1.

**Reviewer 2.10:** Line 157: why can people other the reviewers not access this. I would like to encourage the authors to add them.

**As a response**: The reason others can't access the protocol is because it is not published yet (i.e. still as a work in progress during the manuscript preparation phase). We can publish the protocol, after which it will be publicly available permanently (after a 1-2 week review time on Protocols.io). Meanwhile, we have also added the protocol as a Supporting information to the manuscript.

**Reviewer 2.11:** Line 165: there is systematic bias in the study design (see general comment)

**As a response**: Here we refer to our previous response above and have added comments on the effect of a possible clearance related to *A. lumbricoides* and *T. trichiura* to the Discussion on lines 499-505 and 551-552

**Reviewer 2.12:** Line 177: this exclusion of samples also needs to be discussed; in a way you are favoring for clear slides.

**As a response**: We refer here to the previous response.

**Reviewer 2.13:** Line 193: how long does it take to scan one slide

**As a response**: This was also requested by Reviewer 1 and information has been added to the manuscript. Scanning the entire Kato-Katz smear typically takes 5-10 minutes and the information is added on lines 231-233.

**Reviewer 2.14:** Line 212: per slide / all slides

**As a response**: The figures on line 212 in the original manuscript refer to upload time per slide and this was added to the amended version on line 248.

**Reviewer 2.15:** Fig 2: Can you confirm the scanning automated?

**As a response**: This question was also raised by Reviewer 1 and we have clarified on lines 228-231 that after adjusting course focus on a single field of view, the scanning is fully automated.

**Reviewer 2.16:** Lines 223 – 232: this might require a review by an AI-expert

**As a response**: Based on the comments provided by the Reviewer 1, we assume that the reviewers have the expertise to assess the described method and that it complies with the STARD-AI guidelines (i.e. computational environment, description of software, version of deep learning algorithm, hardware used for training etc) and represents a state-of-the-art method used in a large number of previous studies.

**Reviewer 2.17:** Lines 245: not clear whether all digitized slides (line 245) or only discrepancies (line 186-189)

**As a response**: We have now clarified that annotations mentioned on line 245 in the original manuscript were made in the training set and discrepancies mentioned on lines 186-189 concern the test set on lines 289-290 and 294-296 in the new manuscript

**Reviewer 2.18:** Fig 4: see remark on definition, of ground truth

**As a response**: Here we refer to a previous response above and have changed the term “ground truth” to “reference standard” throughout the manuscript

**Reviewer 2.19:** Line 277: can one indicate the number of slides per STH specfies; per set (training, valiation).

**As a response**: We have specified the number of slides in which annotations were made for training of the algorithm, for each species respectively on lines 300-302. The number of slides per species for the test set are reported in Table 1.

**Reviewer 2.20:** Line 294: please define the level of confidence

**As a response**: The term "level of confidence" refers to the degree of certainty or trustworthiness that a machine learning model assigns to its predictions and corresponds to a probability score. It is a measure of how confident the model is in the accuracy of its output. This text has been added to line 338.

**Reviewer 2.21:** Statistical analysis: Line 309: The word ‘comparable’ suggests an equivalence testing

**As a response**: As also commented on related to the same question raised by Reviewer 1, we have now specified that we used the Buderer formula for equivalence of diagnostic tests and specified this on lines 355-360.

**Reviewer 2.22:** Line 314: not sure where the correlation refers to; I am assuming EPG; see also general comment.

**As a response**: As a response, it has been confirmed on lines 365 that it refers to EPG

**Reviewer 2.23** Results: See general comments on how results are represented. Would welcome more alignment with the hypothesis (‘comparable to the ground thruth).

**As a response**: Here we refer to our response to the general comments above and the changes made accordingly.

**Reviewer 2.24:** Table 3. Replace AL by Ascaris lumbricoides

**As a response**: This was also pointed out by Reviewer 1 and has been changed accordingly.

**Reviewer 2.25:** Discussion : Line 400 – compared to the ground truth (manual microscopy) I would argue that the accuracy is not high; of course reviewing the discrepancies it does. Again, it might help to re-define ground truth.

**As a response**: Here we refer to our previous response and the Supporting Table 1 added with the re-defined reference standard (ground truth).

**Reviewer 2.26:** Line 448 – perhaps it would be good to emphasize a health centre looks like; is indeed representative for a setting with poor infrastructure

**As a response**: We have added a link to the hospital website <https://www.kinondokwetuhospital.com> that shows pictures of the facility. The hospital is fully owned and supported by a trust fund (Kinondo Kwetu Trust Fund) and serves the Kinondo region in Kwale County and provides basic health care, treatment of malaria, HIV, TB, parasitic diseases, antenatal care, deliveries, child care, vaccinations and health information. The laboratory has basic laboratory equipment including light microscopes, hematology, biochemistry and CRP analyzers, as well as rapid test kits. This information has been added to the Study Design on lines 150-156.

**Reviewer 2.27:** How do the authors feel about the extrapolation of the findings to other setting

**As a response**: We have added a comment to the Conclusion on lines 597-598 that the method could be applicable also in field settings, and that further studies should focus on generalizability and multi-centre validation of the method.

**Reviewer 3.**

**Reviewer 3.1:** Overall, the manuscript adds value to the literature for the detection of eggs of STH in developing countries by providing confidence of removing skilled personnel for microscopy. The methods are appropriate, results are presented nicely and interpreted carefully. Therefore, I recommend this paper for publication after a minor revision due to the following reasons:
Abbreviations used in some tables need to explained in footnotes.

**As a response**: This was also pointed out by the other reviewers and abbreviations have been addressed accordingly.

**Reviewer 3.2:** Scale bars should be given for microscopic images

**As a response:** Scalebars have been added to all images Scale bars: Fig 3. (a) 5 mm, (b) 50 µm, (c) 25 µm Fig 5. 25 µm, S1 Fig. 25 µm S2 Fig. 25 µm

**Reviewer 3.3:** References should be checked for style and formatting as per the journal's guidelines.

**As a response:** We have checked the style and formatting and used the Style file recommended by the Journal https://endnote.com/style_download/plos-public-library-of-science-all-journals/

**Reviewer 3.5:**: The methods used in this paper are appropriate.

**Reviewer 3.6:**: Results are clearly presented, and they correspond to the aims of this study.

**We thank all the reviewers for their valuable comments and effort to review the manuscript**.
