# Supplementary material for: Diagnosis of soil-transmitted helminth infections with digital mobile microscopy and artificial intelligence in a resource-limited setting
Source: PLoS Negl Trop Dis. 2024 Apr 11;18(4):e0012041. doi: 10.1371/journal.pntd.0012041 (PMC11008773; doi:10.1371/journal.pntd.0012041)
Supplement: Attachment — Submitted filename: Letter of response Lundin et al revision2_060224.docx [file pntd.0012041.s009.docx]

**Point-by-point response related to the manuscript “Diagnostics of soil-transmitted helminths with digital mobile microscopy and artificial intelligence in a resource-limited setting” (PNTD-D-23-00949)**

We thank the reviewers for a careful assessment of our manuscript, and we have addressed all issues raised point-by-point below. Line numbers refer to the manuscript version “Lundin_et_al_track_changes_060224”

**REVIEWER 1**

Reviewer #1: The objectives of the study are clearly articulated. Further comments are included in the attachments.

Lundin et-al, reports a digital mobile microscopy integrated with Artificial Intelligence for the quick detection of stool parasites. Soil Transmitted Helminths is indeed a big challenge in Low Medium Income Countries. Affordable, easy-to-use diagnostic tools would significantly contribute to the control and elimination programs. The paper proposes a digital imaging platform that is superior in performance to the reference manual microscopy commonly used. The reported accuracy for detection of STHs with the Deep Learning System is promising.

The submitted manuscript is well written and quite readable as well. However, a few remark that could potentially improve the quality of the paper is stated below:

If these issues are addressed, I recommend the paper for acceptance.

**As a response:** We thank the reviewer for the constructive and positive comments.

Reviewer #1: This seems to me like a piece of engineering work. I struggle a bit to find the scientific contribution of this work. Several research groups are working on developing AI-based digital pathology for Neglected Tropical Diseases. While the introduction highlights the application of image-analytical methods to microscopy diagnostics, the scientific challenge/gap is still unclear. Previous work by Ward et-al is highlighted below. I will expect a clear contribution of the authors work to existing similar approach.

Ward P, Dahlberg P, Lagatie O, Larsson J, Tynong A, Vlaminck J, et al. (2022) Affordable artificial

intelligence-based digital pathology for neglected tropical diseases: A proof-of-concept for the detection of soil-transmitted helminths and Schistosoma mansoni eggs in Kato-Katz stool thick smears. PLoS Negl Trop Dis 16(6): e0010500. https://doi.org/10.1371/journal.pntd.0010500

**As a response:** We clarified even in more detail that although some previous studies like the one by Ward et al analysed the accuracy of AI-based analysis on the object level (parasite eggs), to our knowledge the diagnostic accuracy on a sample level has never been assessed based on digital scans of entire microscope slides, so called whole-slide images (WSIs). We believe that assessment of clinical diagnostic accuracy of AI-based methods requires the analysis of whole slide images (i.e., the entire slide) and reporting of results on a sample-level diagnosis as in our study, to be comparable to current diagnostic methods. This clarification was added to the text in the Introduction on lines 115-116 and in the Discussion on lines 519-526.

Reviewer #1: The equipment and setup used for the digitization of microscopy slides in a primary health care setting listed a laptop computer used to operate the whole slide scanner and manage digital slides. I wonder why an online computational procedure is necessary. The laptop should have sufficient computational capacity to implement the developed AI models offline. This will deal with the challenge of the availability of mobile data networks in remote areas where such devices are needed the most. Authors motivate and justify why the DLS is not implementable on the laptop which is already part of the equipment set-up. A deepLabV3-MobileNetV3 deep learning model has been implemented on a Raspberry Pi 4B with Coral USB accelerator as reported by Oyibo et-al. This further re-emphasize the endless possibilities of implementing DLS on general computers. An online DLS systems does not seem to add value except the authors could justify this in their work.

**As a response:** We agree that local analysis is an attractive solution, but might not be feasible with the method described Oyibo et al. In the mentioned study on deep learning based detection of Schistosoma haematobium in urine samples, an algorithm was developed that was run locally and reached a high accuracy on an image (e.g. tile or field-of-view) level. The computational speed was approx. 10 images per minute (sized 1,520 x 2,028), equivalent to 0.5 megapixels second as compared to approx. 14 megapixels per second with our method (2500% faster). This means that analysing one of our WSIs with the described local solution would take almost 2 hours, as compared to 15-25 minutes (upload 10-20 minutes + analysis 5 minutes) with our cloud solution. This was added to the Discussion on lines 591-598. We also already elaborated on some other advantages of a cloud-based solution in the Discussion on lines 584-588.

Reviewer #1: How many fields of view are required to cover the entire sample. I can imagine that with a 20x objectives, there is gain in resolution but decrease of the field of view. This would translate to the need for more images and increased processing time as well. Author should comment on why a low magnification is not used?

**As a response:** As stated in our previous response letter, the microscope vendor does not disclose exact information on the field of view, scanning parameters, or the overlap of adjacent images during scanning; therefore, it is not clear how many images are recorded. However, digitizing a sample with a size of 25 mm x 25 mm, using a microscope equipped with a 6-megapixel camera and an optical system resulting in a pixel size of 0.48 um/px on the image plane, would require a minimum of approximately 450 images. In practice, the number of images is larger when considering the overlap needed for stitching and the possible cropping of the maximum field of view available to retain better image quality across the field of view. On the cloud server the WSI is stored as smaller (512 x 512 pixels) image tiles and a typical sample consists of approximately 10,000 tiles. Information on this is given on lines 340-343.

We used the standard magnification of 20x that most of the microscope scanners on the market uses. A lower magnification might be enough to detect the parasite eggs, but since we aim for a multipurpose solution e.g. useful also for other diagnostic purposes such as screening for pre-cancerous cells in pap smears (Holmström et al. JAMA Network Open 2021;4(3):e211740-e) we decided to use the current instrument. We added a comment on this to the Discussion section on lines 608-612.

Reviewer #1: The end-to-end sample scanning, upload and processing time is still unclear. Author should clearly state the total time from sample insertion to result output. Is there a gain in time as compared to reported existing techniques. There are existing offline methods that proposes a turnaround time of 15 minutes. What is the value addition of this work in that regard?

**As a response:** To clarify this, we added comments on the total turnaround time to the Discussion section on lines 600-605. We also added some comments on how to make the turnaround time faster.

Reviewer #1: Data annotations requires some expert opinion, are the researchers experts in this field? Could this be well stated?

**As a response:** We now clarified that the initial annotations were done by the researchers (AS, OH, SH, SV), after thorough training on the morphology of the STHs provided by the laboratory technologist (FK) who performed the manual microscopy on lines 283-285. Three of the annotators are medical doctors, with previous training on microbiology, cytology and histology.

Reviewer #1: An analysis speed of approximately 14 digital samples per hour seems to be quite slow. What is the plan to increase the processing speed of the samples?

**As a response:** A paragraph on potential ways to improve the speed further was added to the Discussion section on lines 600-612.

Reviewer #1: The parameters of the equations provided in line 346 and 347 are not clearly explained. Authors should explain these parameters accordingly.

**As a response:** This was also pointed by the other reviewer and parameters have been clarified in the Statistical Methods section.

Reviewer #1: What is the limitation of the study?

**As a response:** Limitations (weaknesses) of the study have been elaborated on in the Discussion on lines 553-612. We also changed the word weaknesses to the word limitations. A paragraph on turnaround time was also added, as mentioned above.

**REVIEWER 2**

**Reviewer #2:** Lundin and colleagues have thoroughly revised the manuscript and addressed all my major comments. I only have a few minor comments suggestions that the authors may want to consider to further improve the manuscript. 

Abstract
Line 21: there is a conflict in words; NTDs refers to diseases, while soil-transmitted helminths refer to the pathogens; better to use ‘soil-transmitted helminthiasis’ instead

**As a response**: We thank the reviewer for pointing this out and have changed the wording to ‘Infections caused by soil-transmitted helminths are the most prevalent neglected tropical diseases..’

Line 24: WHO target is not to eliminate STH, rather the elimination of STH as a public health problem.

**As a response**: The wording was changes according to the suggestion

Line 47: I think there is broad consensus that this not perfect. So I am not sure whether you can make this conclusion.

**As a response:** The part of the conclusion stating ‘which challenges the role of manual microscopy as gold standard for detection of STHs’ was removed.

Author summary
Line 53: stool instead of feces

**As a response:** This was changed accordingly

Introduction:
See previous comment on diseases vs. pathogen

**As a response:** We modified the text accordingly e.g. ‘The most prevalent NTDs are **infections caused by** the soil-transmitted helminths (STHs)’ and ‘Together **these pathogens** cause chronic infections’

Line 73: they are all roundworms, Ascaris is sometimes referred as giant roundworm

**As a response**: We changed the wording accordingly

Line 83: prefer to state ‘recommended method’ or ‘current diagnostic standard’ instead of ‘gold standard’

**As a response:** ‘gold standard’ was changed to ‘recommended method’

Line 96: FECPAKG2 instead of FECPAK (this the first generation)

**As a response**: We changed the wording accordingly.

Line 97: not all of these have a higher sensitivity.

**As a response**: We changed the wording to ‘typically higher sensitivity and specificity’

Line 118: ‘Kato-Katz thick smear’ instead of ‘faecal samples’

**As a response**: We changed the wording accordingly.

Line 119/line218: ‘smears’ instead of ‘samples’; check throughout the manuscript

**As a response**: We changed the Kato-Katz samples to Kato-Katz thick smears throughout the manuscript

Methods
Lin 161:

**As a response**: Line break added

Line 171: mL instead of ml

**As a response**: Changed accordingly.

Line 274: n = 388?

**As a response:** We confirm that this number is correct

Line 264: Fig 3 instead of Figure

**As a response**: Changed accordingly.

Line 343: what does epsilon means in these formulae?

**As a response**: Epsilon is the precision parameter, abbreviations added also for the other parameters in the formula.

Table 2: omit the row for deep learning; this is made clear in the caption

**As a response:** The line was omitted
Line 405: Table S1 instead of S Table 1

**As a response:** Changed accordingly
Line 407: Fig S1 instead of S1 Figure

**As a response:** Changed accordingly

Lines 437 – 441: the core question is whether the classifications of infection intensity based on DLS would be different when using manual microscopy. This is more relevant than the difference in mean in EPG. I would like to see this in the body of the text instead of putting into it in supplementary info

**As a response:** We created a new Table 5 showing the classification of the STH infections according to intensity classes. Also, we added some comments on the new Table for to the body text on lines 450-460.

Discussion 
Line 453: avoid the use of gold standard

**As a response:** We changed the wording to ‘recommended method’ as done also in the Introduction
Line 492: the way forward would be either to randomize the KK slide over manual or WSI; or to prepare a duplicate slide which are then processed in parallel.

**As a response:** We modified the suggestion for future studies accordingly on lines 510-511.

Line 518: put genus names in italic; note that the performance in the TPP are absolute, and not relative to the manual reading of KK, and hence may want to tune down the conclusions a bit down.

**As a response:** Genus names now in italics. We also added the following sentence to the Discussion: “However, the diagnostic accuracy of our method as compared to more sensitive and specific STH diagnostic methods (i.e. closer to a ‘ground truth’), such as PCR-based methods or similar remains to be established.” on lines 544-546.

In addition to the above described changes, we revisited the text and made some smaller edits related to the consistency of terminology (eg STH eggs instead of parasite eggs) and corrected some spelling mistakes, all visible in the track changes version of the manuscript.
